# Therapeutic potential of N-methyl-D-aspartate receptor modulators in psychiatry

**DOI:** 10.1038/s41386-023-01614-3

**Published:** 2023-06-27

**Authors:** Jesse E. Hanson, Hongjie Yuan, Riley E. Perszyk, Tue G. Banke, Hao Xing, Ming-Chi Tsai, Frank S. Menniti, Stephen F. Traynelis

**Affiliations:** 1 Department of Neuroscience, Genentech Inc., South San Francisco, CA 94080 USA; 2 Department of Pharmacology and Chemical Biology, Emory University School of Medicine, Atlanta, GA 30322 USA; 3 MindImmune Therapeutics, Inc., The George & Anne Ryan Institute for Neuroscience, University of Rhode Island, Kingston, RI 02881 USA

**Keywords:** Pharmacology, Ion channels in the nervous system

## Abstract

N-methyl-D-aspartate (NMDA) receptors mediate a slow component of excitatory synaptic transmission, are widely distributed throughout the central nervous system, and regulate synaptic plasticity. NMDA receptor modulators have long been considered as potential treatments for psychiatric disorders including depression and schizophrenia, neurodevelopmental disorders such as Rett Syndrome, and neurodegenerative conditions such as Alzheimer’s disease. New interest in NMDA receptors as therapeutic targets has been spurred by the findings that certain inhibitors of NMDA receptors produce surprisingly rapid and robust antidepressant activity by a novel mechanism, the induction of changes in the brain that well outlast the presence of drug in the body. These findings are driving research into an entirely new paradigm for using NMDA receptor antagonists in a host of related conditions. At the same time positive allosteric modulators of NMDA receptors are being pursued for enhancing synaptic function in diseases that feature NMDA receptor hypofunction. While there is great promise, developing the therapeutic potential of NMDA receptor modulators must also navigate the potential significant risks posed by the use of such agents. We review here the emerging pharmacology of agents that target different NMDA receptor subtypes, offering new avenues for capturing the therapeutic potential of targeting this important receptor class.

## Introduction

**Fig. 1:**
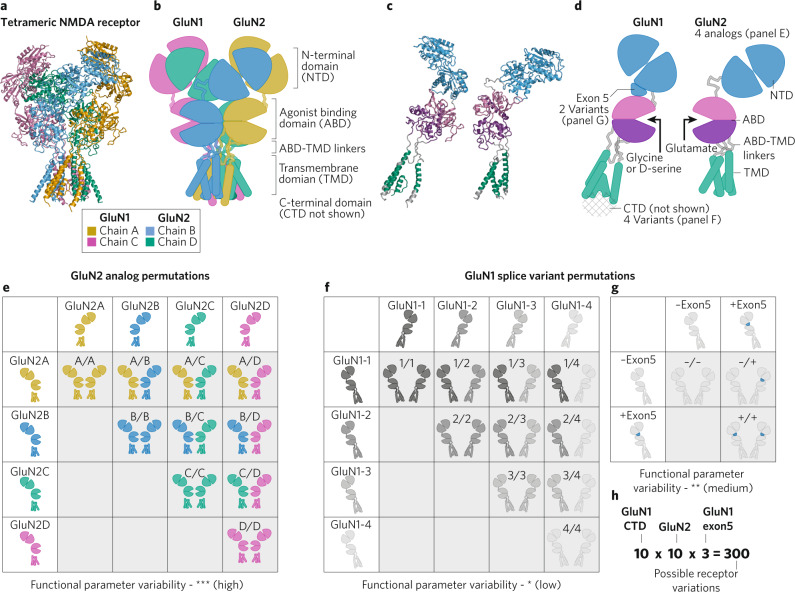
NMDAR diversity arising from assembly of different subunits and splice variants. **a** The NMDAR structure (a model based on pdb: 6WHS) is shown with each protein chain a different color. The C-terminal domain is not present due to the lack of structural data and likely disorder in this region. **b** One copy of each GluN subunits is shown isolated, each domain is highlighted by a different color (NTD, blue; ABD, magenta and purple; linkers portions, grey; TMD, green). **c** Cartoon of the NMDAR highlighting each protein chain (same colors as in **a**). **d** One copy of each GluN subunit is shown isolated as a cartoon diagram, with each domain highlighed by the same colors used in (**b**). Most of the sites of variation are highlighted (GluN2 analogs, GluN1 splice variants). **e**–**g** Permutation tables of the possible combinations of a single NMDAR showing the GluN2 subunits (**e**), the GluN1 CTD splice variants (**f**), and the GluN1 exon5 splice variants (**g**). **h** The total possible permutations of NMDARs is calculated by independent inclusion of the potential variable elements listed in (**e**–**g**).

N-methyl-D-aspartate receptors (NMDARs) are cation-selective ligand-gated ion channels that, together with other ionotropic receptors (kainate receptors, AMPA receptors) and G-protein coupled receptors (metabotropic glutamate receptors, or mGluRs), mediate glutamatergic synaptic transmission throughout the central nervous system [1]. NMDARs are considered to be a principal element of the postsynaptic density, but also are present both presynaptically and extrasynaptically and thus have a broad role in regulating neurnonal excitability and mediating synaptic transmission [2–4]. The classic NMDARs are heteromeric tetramers that comprise two glycine-binding GluN1 subunits (encoded by the *GRIN1* gene) and two glutamate-binding GluN2 subunits (GluN2A-2D, encoded by *GRIN2A-2D*) (Fig. 1a–d) [1]. Each GluN subunit shares a similar architecture that contains four semi-autonomous domains (Fig. 1b, d): an aminoterminal domain (NTD, also known as the ATD), an agonist binding domain (ABD, also known as the ligand binding domain, LBD), a transmembrane domain (TMD) that contains 3 transmembrane helices (M1, M3, M4) and a re-entrant pore-forming loop (M2), and a carboxy-terminal domain (CTD) located intracellularly [5]. GluN1 subunits can also coassemble with glycine-binding GluN3A or GluN3B subunits to form a glycine-activated cation channel, the properties and functions of which are only beginning to be understood [1]. Given that glutamate is the major excitatory neurotransmitter in the central nervous system and NMDARs play critical roles in synaptic activity, it is not surprising that maladaptive NMDAR signaling is implicated in a broad range of neuropsychiatric, neurodevelopmental, and neurodegenerative disorders [6]. Thus, modulation of NMDARs has broad therapeutic potential. This review will focus on recent therapeutically-relevant advances in pharmacological modulation of the glutamate-activated NMDARs comprising GluN1 and GluN2 subunits.

## NMDA receptor functional diversity

The activation of NMDAR channels begins with concurrent binding of glycine to GluN1 subunits and glutamate to GluN2 subunits [7]. Agonist binding promotes closure of the bilobed clamshell-like ABDs, triggering conformational changes that propagate to the TMDs via the movement of linker regions connecting the ABD to the TMDs. This leads to movement of the M3 transmembrane helix, which forms the crossing helical bundle that is the channel gate, allowing Na^+^ and Ca^2+^ to enter the cation-selective pore and flow into cells down their electrochemical gradients [8, 9]. The NTDs serve as subunit-specific regulatory units that have intrasubunit allosteric effects on agonist binding, channel gating, and also have the ability to bind and transduce the activities of some extrinsic allosteric modulators [1]. The CTDs anchor the NMDARs to the intrasynaptic cytoskeletal network, contain sites for posttranslational modifications, and serve as scaffolds for binding of signal transduction molecules such as CamKII [1].

NMDAR activation triggers an elevation of intracellular Ca^2+^ concentration and membrane depolarization that contributes to a slow component of excitatory synaptic transmission [1, 10]. A unique aspect of the NMDAR pore is its sensitivity to voltage-dependent Mg^2+^ block that results in channel occlusion at resting membrane potentials, and which is relieved with depolarization. Thus, Mg^2+^ block has been considered a mechansim for Hebbian coincidence detection, linking co-activation of presynaptic (glutamate release) and postsynaptic (membrane depolarization) elements to instigate modification of synaptic efficacy through altered AMPA receptor number and function [1, 11, 12]. In addition to cation channel activity, there is a growing body of evidence indicating that NMDARs also signal via metabotropic mechanisms independent of channel activity in both physiological and pathological conditions [13–18]. Thus, NMDARs can tranduce a multifaceted signal tuned to the requirements of specific circuitry and individual synapses.

NMDAR functional diversity arises from the assembly of receptors with different GluN2 subunits (Fig. 1c–h) that confer different biophysical properties including sensitivity to Mg^2+^ block, sensitivity to allosteric modulators, deactivation time course, single channel conductance, channel open probability for an agonist-bound receptor, and agonist potency (Fig. 1d, e). The potency of Mg^2+^ for voltage-dependent inhibition of GluN2A- and GluN2B-containing NMDARs is nearly 5-fold higher than for GluN2C- and GluN2D-containing NMDARs (Mg^2+^ IC_50_ at −100 mV were 2.4 & 2.1 µM for GluN2A and GluN2B respectively, while 14.2 & 10.2 µM for GluN2C and GluN2D respectively) [10, 19, 20]. GluN2 subunit composition also impacts the deactivation time course, which defines the duration of the excitatory postsynaptic currents mediated by NMDARs [21]. The deactivation time constant for GluN2D-containing NMDARs is 10–100 fold slower than that observed for GluN2A-containing NMDARs (1000–5000 ms vs. 50–100 ms), while the GluN2B- and GluN2C-containing NMDARs display an intermediate deactivation time constant (i.e., 300–500 ms) [10, 22]. Different GluN2 subunits also impart distinct single channel properties, with GluN2A- and GluN2B-containing NMDA receptors having higher channel conductance levels than GluN2C- and GluN2D-containing receptors [1, 10]. In addition, the NMDARs have an over 50-fold range of variation in channel open probabilities, with values of ∼0.5 for GluN2A-, ∼0.1 for GluN2B-, and 0.01–0.04 for GluN2C- and GluN2D-containing NMDARs [23–26].

GluN2A- and GluN2B- containing NMDARs have a lower glutamate (EC_50_ 3.0–5.0 µM), glycine (EC_50_ 1.0–1.5 µM) and D-serine (EC_50_ 0.7–1.3 µM) potency than GluN2C- or GluN2D-containing NMDARs (glutamate EC_50_ 0.2–0.4 µM; glycine EC_50_ 0.1–0.2 µM; D-serine EC_50_ 0.2–0.3 µM) [1, 10, 27, 28]. Thus, brain levels of glycine and D-serine (5 and 1 μM, respectively; [29]) as well as CSF levels of glycine (7–10 μM) and D-serine (1–3 μM; [30–32]) suggest the glycine site is not saturated for GluN2A- and GluN2B-containing NMDARs. By contrast, these levels of glycine and D-serine are over 5-fold the EC_50_ at GluN2D-containing NMDARs, indicating that preferential regulation of GluN2A and GluN2B receptors might be possible by increasing extracellular glycine or D-serine. If, say 70% of NMDARs are glycine-bound, a modest ~43% potentiation is achievable by increasing glycine or D-serine concentrations (43% potentiation = 100%/70%). Direct treatment with glycine-site agonists [33–36] and a related strategy to increase glycine through inhibition of a glycine transporter [37] have been explored clinically.

In addition to diheteromeric NMDARs consisting of GluN1 and a single type of GluN2 subunit, triheteromeric receptors that contain two different GluN2 subunits are also formed. It appears that the functional parameters of triheteromeric receptors can be dominated by one of the subunits, particularly GluN2A, or have properties intermediate to the those of the two GluN2 subunits, for example GluN2B/GluN2D triheteromers [7, 38–46]. Furthermore, the selectivity and activity of pharmacological agents can also be distinct in the context of a triheteromeric complex.

Another source of functional variation is splice variants of the GluN1 subunit (Fig. 1d, f, g). There are 8 different GluN1 splice variants, which arise from alternative splicing of 3 different exons (exon 5, 21, and 22), and it is possible for NMDARs to include two different GluN1 splice variants in a single receptor complex [45]. These GluN1 splice variants confer differences in glutamate and glycine potency, deactivation rates [26, 45, 47], and regulation by endogenous modulators such as Zn^2+^ and protons [48, 49]. In addition, alternative splicing can alter synaptic plasticity [50]. There are also important variations conferred by differences in the intracellular CTDs that link NMDA receptors to a multitude of different intracellular signaling complexes [1]. For example, the CTD of the GluN2B subunit uniquely has docking sites for CaMKII, which is thought to confer specificity to GluN2B-containing NMDARs in activation of this kinase to trigger modification of synaptic strength. It was recently discovered that GluN2A is also phosphorylated by CaMKII, leading to altered protein interactions, decreased surface expression and reduced synaptic function [51, 52]. In addition to GluN1 splice variants, a primate-specific GluN2A splice variant has been described that may confer additional functional diversity [53].

The potential variation in receptor composition arising from different GluN2 subunits and different GluN1 splice variants yields a dizzying number of different NMDARs. Across the CNS, if one considers combinations of all of the four GluN2 subunits with the inclusion or exclusion of exon5, exon21, and/or exon22 in GluN1, there may be as many as 300 unique NMDA receptors exhibiting a continuum of properties (Fig. 1h), although no specific neuron will show this full range of diversity given that NMDARs are differentially expressed in different cell types at different times. NMDARs with distinct properties and regulation mechanisms are differentially deployed to serve the function of particular circuits, neurons, and synapses. Modulators that are selective for NMDARs of different subunit composition are becoming increasingly available [1], offering the possibility of targeting therapeutic intervention to specific circuitry based on subunit distribution.

## Distribution of NMDA receptor subtypes

**Fig. 2:**
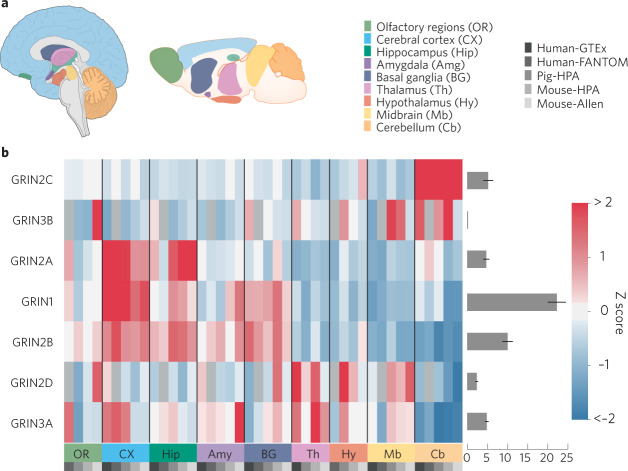
Brain region expression patterns of NMDAR subunits. **a** Overview of brain regions where expression data were compared and data sets used in the analysis. All images and data were modified from the Brain section of the Human Protein Atlas (proteinatlas.org). Data for distribution of expression levels were derived from quantification of mRNA levels from RNAseq and in situ hybridization experiments as well as protein levels from immunohistochemistry and immunofluorescence experiments [234]. Data was downloaded from v20.proteinatlas.org and was reorganized and replotted with custom python scripts using pandas, matplotlib and seaborn libraries without further data processing. **b** Heatmaps show relative expression levels for NMDAR subunits. *Z*-scores reflecting relative expression across brain regions were calculated separately for each gene. Relative expression levels (averaged across brain regions) of each subunit compared to other subunits are plotted to the right of each heatmap.

**Fig. 3:**
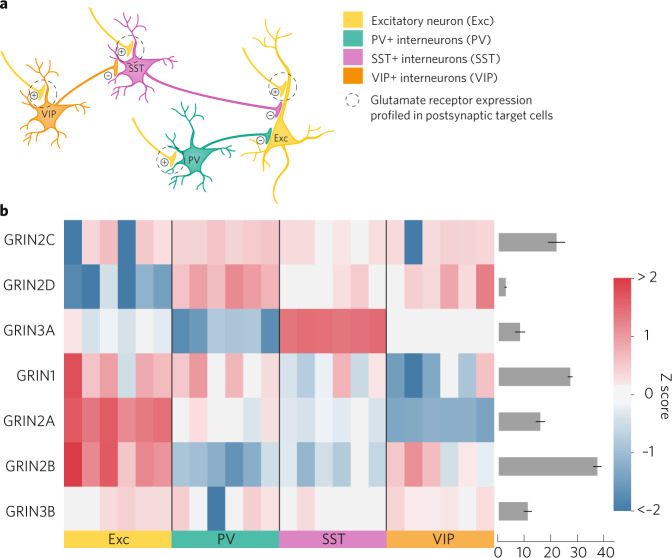
Neuronal expression patterns of NMDAR subunits in a canonical cortical/hippocampal microcircuit. **a** Simplified diagram of the canonical cortical/hippocampal circuit consisting of excitatory pyramidal neurons and three classes of inhibitory interneurons. Only select connections are illustrated. Whereas synaptic locations of postsynaptic glutamate receptors are illustrated, expression data comes from profiling isolated cell bodies of each neuron subtype [77]. Neuron subtype gene expression data from [77] (GSE122100) were plotted for the brain region plots as in Fig. 2. **b** Heatmaps show relative expression levels of the different NMDAR subunits in the cortical/hippocampal cell types. Each column represents an individual sample (mouse) and *Z*-scores reflecting relative expression across samples were calculated separately for each gene. Expression levels (averaged across all samples) of each subunit are plotted on the bar graph to the right of each heatmap.

NMDAR subtype distribution may be considered at the macroscopic circuit level (Fig. 2) or at the level of distribution within the neurons of specific circuits (Fig. 3). The GluN1 subunit is ubiquitous in expression throughout the central nervous system, with GluN1 splice variants having regional and developmentally specific distributions [54]. The four GluN2 subunits also show distinct temporal and spatial expression profiles [55–59]. GluN2B and GluN2D are the first GluN2 subunits to be expressed at early embryonic and neonatal stages, with the GluN2A and GluN2C subunits beginning to be expressed after birth [57, 60]. Pharmacological agents that show clear selectivity between diheteromeric subtypes, those containg two of the same GluN2 subunits, have been essential tools to confirm expression of functional NMDAR subtypes. However, the use of these agents still presents challenges given presence of triheteromeric NMDARs containing two different GluN2 subunits [7].

In the adult brain, GluN2A and GluN2B are broadly expressed across many regions, with especially high expression in forebrain in principal neurons (Fig. 2). Functional evidence for the expression of synaptic GluN2A and GluN2B has been inferred from deactivation parameters and the sensitivity to GluN2B-selective NAMs [61–63]. The recent GluN2A-selective NAMs, TCN-series and MPX-series, are the first pharmacological agents with sufficent selectivity to be useful to probe for functional GluN2A-containing NMDARs [64, 65]. Some earlier studies used the competitive antagonist NVP-AAM007 (also known as PEAQX) for this purpose, but this agent did not have sufficient selectivity for conclusive experimentation [66, 67]. Newer competitive antagonists are becoming available with better selectivity to discriminate GluN2A from GluN2B [68]. There is now clear evidence for the deployment of synaptic GluN2A/GluN2B triheteromers along with GluN2A and GluN2B diheteromers [46, 69, 70]. Receptor composition is dynamic as synapses cycle through levels of higher or lower ratios of GluN2B- to GluN2A-containing receptors, which holds implications for NMDAR-dependent synaptic plasticity [71, 72].

Localization and functional studies indicate that GluN2A- and GluN2B-containing NMDARs are also present in the perisynaptic membrane. These receptors may be activated by extrasynaptic glutamate to play a role in regulating membrane potential and synaptic excitability. GluN2B-containing NMDARs are more mobile than GluN2A-containing NMDARs [73], and early studies suggested GluN2B-containing NMDARs comprise a greater proportion of the extrasynaptic NMDAR pool [74–76]. However, more recent studies indicate that both GluN2A- and GluN2B-containing NMDARs exist extrasynaptically and that the ratio of the two subtypes is close to that of synaptic NMDARs [2–4].

GluN2A- and GluN2B-containing NMDARs are differentially expressed by forebrain interneurons (Fig. 3). Among inhibitory interneurons, GluN2A has relatively higher expression in medial ganglionic eminence-derived interneurons expressing parvalbumin (PV) and somatostatin (SST), while GluN2B has relatively higher expression in regular spiking, central ganglionic eminence-derived interneurons that express vasoactive intestinal peptide (VIP) ([77–80], Fig. 3). Potenitally consistent with this expression pattern, NMDAR responses in regular-spiking radiatum interneurons were found to be senstive to inhibition by the GluN2B NAM Ro 25-6981, whereas this compound had no effect on responses in fast-spiking, presumabley PV expressing, neurons in the hippocampus [81]. In interneurons, GluN2A and GluN2B maybe co-assembled as triheteromers with GluN2D [82].

While broadly expressed in early development, GluN2D expression decreases with age, becoming localized to several regions, including thalamus, basal ganglia, and GABAergic interneurons throughout the brain and spinal cord [1, 10, 57, 58, 60, 83–88]. GluN2D mRNA is detected in PV-, neuropeptide Y- and SST-positive interneurons classified either as fast- or regular-spiking [60, 89, 90], with expression levels highest in PV-positive interneurons (Fig. 3). Recent functional studies utilizing subtype-selective pharmacology support GluN2D expression in cortical and hippocampal interneurons [46, 82, 89–92], striatum [93–96], subthalamic nucleus [69, 82, 97], substantia nigra [98–100], cerebellum [83, 101] and spinal cord [102, 103]. GluN2D can co-assemble with GluN2B, as has been shown in subthalamic neurons [69]. It is not know whether GluN1/GluN2D diheteromeric NMDARs are expressed in native tissues [39, 40, 97, 104], with only a single example of responses with temporal properties compatible with NMDARs that contain two copies of GluN2D [105].

GluN2C subunit expression is highest in cerebellum, anteroventral and dorsolateral thalamic reticular nuclei, other thalamic nuclei, and olfactory bulb, with only weak expression in the cortex, hippocampus, striatum, amygdala, and spinal cord (Fig. 2). The exception is strong GluN2C mRNA labeling in layer I of the cortex, which may reflect expression in glial cells [60]. Functional studies utilizing GluN2C-selective pharmacology support neuronal expression in the cerebellum, thalamus, and globus pallidus [44, 106–108]. GluN2C is also expressed in astrocytes [57, 60, 109–112] as well as oligodendrocytes [60, 113–117] in forebrain regions. GluN2C appears to coassemble with GluN2A, whereas there is some evidence indicating that GluN1/GluN2C diheteromeric receptors are not expressed in native tissues [44, 97, 118].

Overall, the differential distribution of NMDARs subtypes provides an opportunity for subunit-selective modulators to have unique effects on specific circuits. The therapeutic potential of this consequence is discussed in the next section.

## NMDARs as a therapeutic target

NMDAR signaling is essential for normal brain functions, from neuronal development, to sensory/motor integration, to learning and memory [1]. Accordingly, pertubrations in NMDAR function are implicated in a wide range of neurological conditions (e.g., epilepsy/seizures, stroke and traumatic brain injury, Alzheimer’s and Huntington’s disease, neuropathic pain), neuropsychiatric conditions (e.g., depression, schizophrenia, addiction, anxiety), and neurodevelopmental disorders (e.g., autism) [1, 6, 10, 84, 119–124]. Given the physiological roles and implication in pathology, NMDARs are targets for development of therapeutics across the spectrum of CNS diseases and disorders. At the same time, given their critical roles in normal physiology, NMDAR modulators are fraught with the potential for adverse effects. In human use and in non-human preclinical studies, NMDAR modulators may disrupt sensory/motor integration, cognitive functions and, in some cases, cause neuronal damage [123–125]. Thus, choosing the pharmacology and optimizing the use of NMDAR modulators needs to be done prudently if success is to be achieved in capturing the therapeutic potentials of such agents while maintaining safety.

**Fig. 4:**
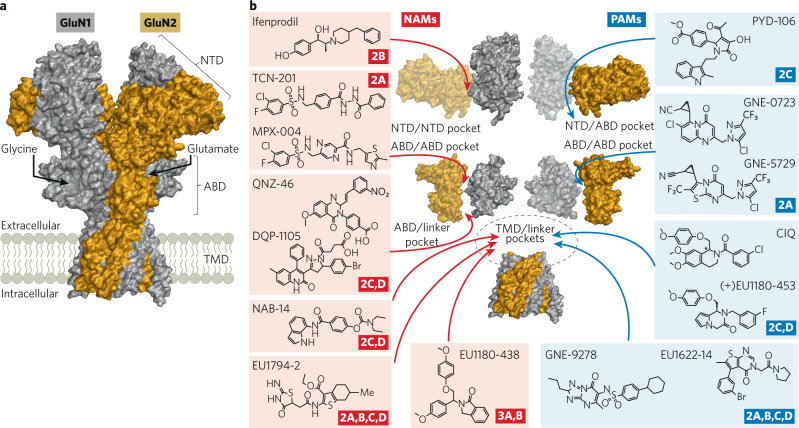
Site of action of NMDAR allosteric modulators. **a** Space-filling representation of a GluN1/GluN2B structure (model based on PDB:6WHS [235]) where *Gray* is GluN1 and *Gold* is GluN2B. **b** Hypothesized site of action for NAMs (red) and PAMs (green) is shown along with subunit selectivity. Data supporting the sites of action include mutagenesis studies from QNZ-46 [236]; DQP-1105 [237]; NAB-14 [82]; EU1622-14 [197]; EU1794-2 [198]; EU1180-438 [238]; CIQ [136, 239]; ( + )EU1180-453 [225]; GNE-9278 [199] and structural studies for ifenprodil [8]; TCN-201, MPX-004 [141]; GNE-0723, GNE 5729 [210]; PYD-106 [142, 143].

The decades-long history of waxing and waning interest in the therapeutic potential of NMDAR modulators has been extensively reviewed [126–131]. The past decade has been one of waxing interest, driven by two factors. The first is the success of the NMDAR antagonist ketamine as a rapid acting, highly effective antidepressant. To quote Niciu et al “*Ketamine’s rapid antidepressant effects has been viewed by some experts in the field as arguably the most important psychiatric discovery in half a century*” [132]. The second is the emergence of new pharmacologies that target specific NMDAR subtypes. The prototype subunit-selective agents are the GluN2B NAMs, development of which was catalyzed by Keith Williams’ finding of the GluN2B selectivity of ifenprodil in 1993 [61]. More recent identification of GluN2A-selective NAMs [64], GluN2A-selective PAMs [133], and GluN2C/D-selective PAMs and NAMs [134–136] provides a new toolbox (Box 1, Fig. 4) with which to ask questions about the receptor subtypes and circuits that underlie different brain functions and contribute to neuropathology. The field of NMDAR pharmacology has been significantly facilitated by structural studies [137–140] that continue to reveal the complexity of NMDA receptors, including the differences imparted by the different GluN2 subunits and mechanisms underlying allosteric modulation [8, 141–143]. In the following sections we will first discuss what ketamine and the GluN2B-selective NAMs are revealing about the pathology underlying depression and cognitive dysfunction. We then discuss NMDAR PAMs and their therapeutic potential in schizophrenia and other disorders.

Box 1 Subunit-Selective NMDAR modulators bind to multiple sites The first subunit-selective NMDA receptor modulator discovered was ifenprodil, which Keith Williams beautifully showed in 1993 was a selective inhibitor of GluN2B-containing NMDARs almost immediately after cloning of the GluN2 subunits [61]. It would take over 15 years before modulators with different NMDAR subunit selectivity were found. However, in 2010 the dam began to break. Dan Monaghan described an interesting series of NMDAR modulators with a range of differing subunit selectivity [240]. Bettini et al. also described in 2010 the first GluN2A-selective inhibitor [64], which acted by a novel mechanism involving reduction in glycine affinity [241]. Also in 2010, Mullaserril et al. described the first GluN2C/GluN2D-selective potentiator [136], and Mosley et al. described the first GluN2C/GluN2D-selective inhibitor [134], followed closely in 2011 by a second class of GluN2C/GluN2D-selective inhibitors [237]. The wealth of new possibilities described in roughly a single year injected a refreshing breath of life into NMDA receptor pharmacology, and shortly after this period, selective potentiators were described for GluN2A [133] and for GluN2C [226], along with a wide range of inhibitors and potentiators with mixed pharmacology, mixed subunit selectivity, unusual pore-modifying properties, including those based on endogenous neurosteroids (reviewed by [1, 131]). These modulators and their proposed binding sites, shown in Fig. 4, are now being used to uncover unexpected pharmacology of circuits and new roles for different NMDAR subunits. All of this has stimulated creative thinking about ways to tune NMDAR function for therapeutic gain.

### Ketamine, GluN2B NAMs, and depression

The remarkable aspect of the antidepressant effects of ketamine (Box 2) is that they are realized after a brief exposure and then persist in a significant number of responders for days to weeks after the drug is cleared. Thus, the antidepressant response is the result of durable changes in the brain that develop after transient NMDA receptor inhibition, not with sustained inhibition. Studies by many laboratories including the late Ron Duman, Monteggia and colleagues, and others [144–146] indicate that the antidepressant-associated changes relate to an upregulation of BDNF-dependent neurotrophic activity and increases in synaptic efficacy and/or plasticity. There is now significant interest in gaining a better understanding of how brief inhibition of NMDARs by ketamine activates signaling mechanisms that lead to these changes, and which circuitry is critical to the antidepressant response. GluN2B NAMs also produce rapid antidepressant effects (see below), and comparing the effects of ketamine to GluN2B NAMs may help to provide such mechanistic insights.

The initial identification of ifenprodil as a NAM selective for NMDARs containing a GluN2B subunit triggered a rapid expansion of this pharmacological class that continues today [147–150]. Structural and mechanistic studies have yielded a wealth of information about how GluN2B NAMs inhibit NMDA receptor function [8]. Several compounds of note have provided important preclinical and clinical results. Ro 25-6981 and CP-101,606 have been used for decades in preclinical studies [151–154]. Recent preclinical data published on BMS-986169 and BMS-986163 include direct comparisons with ketamine and other GluN2B NAMs [155, 156]. Interestingly, Ro 25-6981, BMS-986163, and CP-101,606 are all reported to mimic those effects of ketamine hypothesized to the underly the antidepressant response (summarized in Table 1). Furthermore, acute administration of GluN2B NAMs impairs performance on cognitive behavioral tasks [81, 157]. Ro 25-6981 and CP-101,606 also cross-discriminate with phencyclidine in drug discrimination paradigms in rats [158, 159] and primates [159] and CP-101,606 is self-administered by primates experienced in self-administration of phencyclidine [159], indicating that some GluN2B NAMs share discriminative stimulus properties with the channel blocker phencyclidine.

**Table 1:** Comparison of the effects of ketamine and GluN2B NAMs in preclinical studies relevant to antidepressant effects and cognitive disruption.

	Ketamine	GluN2B NAMs	References
Functional effects during systemic drug exposure			
Auditory evoked potentials	Inhibition (anesthetic exposures, rat)	No inhibition (rat)	Nagy et al., 2016 [178]
EEG, gamma	Gamma induction (mouse, rat, NHP)	No gamma induction (rat, NHP)	Nagy et al., 2016; Kocsis, 2012; Keavy et al., 2016; Bristow, et al. 2017 [176–178, 228]
Deviance detection, mis-match negativity	Disruption (rat)	Disruption (rat)	Sivarao et al., 2014 [229]
Locomotor activity	Stimulation	No or weak stimulation (compound dependent)	Bristow, et al. 2017; Gilmour et al., 2009 [124, 228]
Drug discrimination	Cross discrimination with GluN2B NAM (rat, NHP)	Cross discrimination with NMDA channel blockers (rat, NHP)*	Chaperon et al., 2003; Nicholson et al., 2007 [158, 159]
Self-administration	Supports self-administration (NHP)	Supports self-administration (NHP)	Nicholson et al., 2007 [159]
Working memory	Disrupts	Disrupts	Weed et al., 2016 [230]
Dissociative-like behaviors	Induces	Do not induce	Bristow, et al. 2017; Weed et al., 2015 [228, 230]
Functional effects emerging after drug exposure			
Auditory evoked potentials	Elevated (rat, subanesthetic exposures)	Elevated (rat)	Nagy et al., 2016 [178]
Hippocampal LTP, ex vivo	Enhanced	Enhanced	Graef et al., 2015 [231]
mTOR signaling	Induced	Induced	Li et al., 2010; Li et al., 2011 [232, 233]
BDNF	Elevated	Elevated	Li et al., 2010; Li et al., 2011 [232, 233]
Synaptic markers	Elevated	Elevated	Li et al., 2010 [232]
Forced swim	Increased swim time	Increased swim time	Li et al., 2010; Li et al., 2011; Bristow et al., 2017 [228, 232, 233]
Novelty suppressed feeding	Reduced feeding latency	Reduced feeding latency	Li et al., 2010; Bristow et al., 2017 [228, 232]
Chronic unpredictable stress	Reduced adverse behavioral effects	Reduced adverse behavioral effects	Li et al., 2010; Li et al., 2011 [232, 233]

Upper rows compare effects during systemic drug exposure relevant to the cognitive disruption and psychotomimetic effects. Lower rows compare effects after the drugs have been cleared relevant to the antidepressant effects. Species are indicated in parentheses, *NHP* nonhuman primate. References are from studies that included both ketamine and a GluN2B NAM. The GluN2B NAMs include Ro 25-6981, CP-101,606, BMS-986169 and/or BMS-986163. *PCP was used in these discrimination studies.

CP-101,606 has been evaluated in several Phase I and Phase II proof-of-concept trials in humans [160]. Of particular interest, CP-101,606 was found to produce a rapid onset antidepressant response that developed after a brief intravenous infusion in patients with treatment resistant depression [160]. Although the study did not compare CP-101,606 directly to ketamine and used a longer timeframe over which to assess depression symptoms, the results were strikingly similar to those reported for ketamine in terms of the magnitude of antidepressant response, the percentage of responders, and the duration of response after the single administration. In the depression study, CP-101,606 also caused cognitive disruption and dissociative effects, as were also observed in a small Phase II study of efficacy against L-DOPA-induced dyskinesias in Parkinson’s disease patients [161]. Very recently, Novartis reported top-line results of a Phase II study of a new GluN2B NAM, MIJ821, in patients with treatment-resistant depression that included ketamine as a direct comparator (NCT03756129). MIJ821 produced rapid onset, robust antidepressant efficacy equivalent to ketamine. The compound also caused mild cognitive impairment that overlapped with those produced by ketamine. Thus, in both human clinical studies and in preclinical studies, ketamine and GluN2B NAMs have strikingly similar profiles in terms antidepressant efficacy, disruption of cognitive function, and dissociative effects. Yet, ketamine and GluN2B NAMs are quite distinct in both their mechanism of action on NMDA receptors and their subunit selectivity.

Box 2 The discovery of ketamine as a fast-acting antidepressant

Ketamine and its anticedent phencyclidine were seminal in the development of therapeutic NMDA modulators [242]. In the 1950’s and 1960’s, Luby and others vividly described the ‘schizophrenomimetic’ effects of phencyclidine in healthy human volunteers [243]. Subsequently linking this activity to NMDAR channel block gave birth to the NMDAR hypofunction hypothesis for schizophrenia [244–248], which is discussed further in the NMDAR PAM section. First investigated as a dissociative anesthetic and analgesic, ketamine gained approval as an anesthetic in 1970 and is still used for this purpose in children and in veterinary practice. Ketamine continues to serve as an experimental probe of NMDA receptor function in heathy subjects and in those suffering from neuropsychiatric disorders. It was such use by Berman, Krystal and colleagues [249] in patients with depression that lead to the discovery of ketamine’s rapid onset antidepressant activity. Ketamine, as the racemate, is now being used off-label to relieve depressive symptoms in patients with inadequate response to standard of care antidepressants, and/or who are experiencing acute, severe depressive episodes. Ketamine is particularly valuable in rapidly ameliorating suicidal ideation and the threat of suicide. Spravato (esketamine), the S-isomer of ketamine, has now gained worldwide regulatory approval for use as an antidepressant. There are excellent reviews of this fascinating story [250–252].

### Mechanisms of ketamine and GluN2B NAMs

Ketamine and the GluN2B NAMs have distinct binding sites and mechanisms by which they inhibit NMDARs. Ketamine blocks the pore of NMDARs irrespective of subunit composition [1], and block is activity-dependent, which confers selectivity for active receptors in functional circuitry. It is estimated that for the commonly used dosing regimen, the antidepressant response to ketamine corresponds to occupancy of approximately 30% of the total NMDAR pool in forebrain [162]. However, block may be concentrated on subpopulations of active receptors that are defined by neuron subtype or network activity [163]. By contrast, GluN2B-selective NAMs bind within the interface of the GluN1/GluN2B NTD dimer to promote the closed configuration of the bilobed-NTDs found in the inactive receptor state. This prevents the agonist-induced conformational changes that alter subunit orientation in both NTD and ABD, which leads to opening of the channel [8]. Two noteworthy features of GluN2B NAMs are that they exhibit activity-dependence and positive cooperativity between glutamate and GluN2B NAM binding [63, 164]. Activity-dependent inhibition could reflect greater access of GluN2B NAMs to their binding site following agonist-induced rearrangements to the NTD dimer [8], however the exact mechanism is unclear. Furthermore, Kemp and colleagues described the remarkable property of GluN2B NAMs potentiating rather than inhibiting NMDAR responses to low submicromolar glutamate [164], an effect not observed for ketamine. Presumably, this reflects an allosteric effect of the GluN2B NAM-bound NTD on the agonist binding domain to increase apparent glutamate affinity, thereby increasing receptor occupancy at low glutamate levels. A potential consequence is that GluN2B NAMs could drive an increase in tonic NMDAR activity to ambient extrasynaptic glutamate, which is in the range of 100 nM [165]. Regardless of the mechanism underlying these properties, they hold important implications for which circuits are most sensitive to GluN2B NAMs and the nature of the effects of these agents on circuit function.

Gaining an understanding of the overlap in the NMDAR populations and circuitry impacted by ketamine and GluN2B NAMs may provide insight into the core mechanisms resulting in the antidepressant response as well as the cognitive disruption and psychotomimetic effects relevant to the NMDAR hypofunction hypothesis of schizophrenia. Two hypotheses have been proposed to account for the antidepressant effects of ketamine. The first is direct inhibition of NMDARs in principal excitatory neurons, which are the locus for the ketamine-induced change in synaptic efficacy that is thought to be involved in the antidepressant response. While principal neurons for the most part are sparsely active, highly active subpopulations have been identified that may have a particularly important role in organizing the activity of cortical ensembles [166, 167]. Both ketamine and GluN2B NAMs could potentially target principal neuron sub-populations that are highly active, given that both ketamine and the GluN2B NAMs are activity-dependent inhibitors. The GluN2B NAMs have greater efficacy at diheteromeric GluN1/GluN2B compared to GluN1/GluN2A/GluN2B triheteromers, suggesting that circuitry dependent on the activity of GluN2B diheteromers may be relevant. In this regard, Arnsten and colleagues identified a population of delay cell persistent firing neurons in prefrontal cortex in which task-related firing is highly sensitive to GluN2B NAMs as well as ketamine [168]. These neurons may instantiate the persistent cortical activity that is the basis for working memory, and so inhibition of NMDAR on these neurons may account for the memory disrupting effects of both ketamine and the GluN2B NAMs. Such GluN2B-dependent neuronal populations may be more broadly involved in psychopathology [169].

A molecular mechanism for the antidepressant actions of ketamine on principal neurons has been proposed by Monteggia and colleagues [144]. In principal neurons, ketamine is proposed to block an NMDAR pool linked to suppression of eEF2K activity. The resulting disinhibition of eEF2K activity leads to an increase in BDNF translation and, in turn, the increase in BDNF signaling supports the sustained change in plasticity underlying the antidepressant response. A tenet of this proposal is that the activity of this NMDAR pool is sustained under basal levels of neuronal activity, with a candidate pool being extrasynaptic NMDARs activated by non-synaptic glutamate. A recent report by Monteggia and colleagues suggested that a key synaptic locus for the antidepressant effects of ketamine is BDNF upregulation at the CA3/CA1 synaptic junction [170]. This framework is compatible with the antidepressant efficacy of GluN2B NAMs. GluN2B-containing NMDARs comprise a significant proportion of the extrasynaptic NMDAR pool [2–4, 74–76] and GluN2B is highly expressed by CA1 pyramidal neurons [60].

Aside from direct effects on principal neurons, a second, hypothesized mechanism for ketamine’s antidepressant effect is an indirect impact on principal excitatory neurons via inhibition of fast spiking PV-positive interneurons resulting in cortical disinhibition [171–173]. Supporting the indirect hypothesis, the high firing rate of PV-positive interneurons, relative to pyramidal neurons and regular spiking interneurons, results in their selective susceptibility to ketamine block due to longer periods of membrane depolarization, less Mg^2+^ block, and more active NMDARs. Consistent with this, in vivo electrophysiology shows ketamine blockade reduces PV-interneuron activity and results in excitatory neuron disinhibition [174]. At the circuit level, reduced PV-interneuron activity is manifest as increased gamma frequency cortical activity, which is hypothesized to account for the acute disruption of cognitive function [175]. However PV neurons are insensitive to GluN2B NAMs [81], and these agents do not alter gamma oscillations in the same manner as ketamine [176–178]. Thus, the PV-interneuron as a locus of action for the induction of psychotomimetic and antidepressant responses does not account for the striking similarity in induction of such responses by ketamine and the GluN2B NAMs. Nonetheless, GluN2B NAMs have been reported to reduce drive to hippocampal regular-spiking interneurons and this may also cause disinhibition of excitatory pyramidal neurons [81]. Thus, it is possible that the effects of the GluN2B NAMs via this alternative interneuron-regulated circuitry may contribute to the cognitive disruption and antidepressant responses, converging further downstream with ketamine-sensitive circuitry. Further insight may be gained by comparing effects of GluN2D NAMs to ketamine and GluN2B NAMs in regard to these questions. The GluN2D NAMs may be predicted to disinhibit PV-interneurons similar to ketamine [90, 179] and so it will be of interest to determine if such compounds similarly impact gamma oscillation, cognitive function, and have antidepressant-like activity.

An additional hypothesis proposes that the antidepressant effects of ketamine may not be due to NMDA receptor inhibition per se but, instead may be due to the action of ketamine metabolites on non-NMDA receptor molecular targets [180, 181]. This hypothesis is difficult to reconcile with the similar clinical efficacies of R/S-ketamine and S-ketamine, which generate different patterns of metabolites, and the GluN2B NAMs, which have chemical structures distinct from ketamine and so have non-overlapping metabolite profiles. More work is needed to understand the role of metabolites in the actions of ketamine [182].

While further research is needed to better understand circuitry and mechanisms, the now decades of experience with ketamine and the GluN2B NAMs in both preclinical and clinical research settings have established two simple but profound observations. The first is that inhibition of NMDARs can have acute deleterious effects on cognitive functions that closely mimic deficits in neuropsychiatric conditions. The second is that inhibition of NMDARs can cause persistent beneficial changes to the brain in the context of depression. Given that NMDAR hypofunction is implicated in conditions with impaired cognitive function, and the above observation that NMDAR inhibition can impair cognition, enhancing NMDAR function with positive allosteric modulators of NMDARs is being pursued as a new approach for the treatment of neuropsychiatric disorders. Progress on the development of such agents is reviewed in the next section.

## NMDAR positive allosteric modulators (PAMs)

Impaired NMDAR transmission has been implicated in a variety of neurological conditions including schizophrenia, neurodegenerative diseases, and epilepsy syndromes. The varying symptoms exhibited across these conditions likely result from manifestation of NMDAR hypofunction in distinct brain regions and cell types in each disease context. In schizophrenia, both human pharmacology and human genetics [183, 184] (*see GRIN2A* section below) implicate NMDAR receptor hypofunction. As described above, treatment with NMDAR antagonists, including ketamine and GluN2B NAMs, can induce the core symptoms of schizophrenia in healthy volunteers and exacerbate symptoms in patients [185–187]. Preclinical studies using pharmacological and transgenic approaches also demonstrate that impaired NMDAR function causes schizophrenia-like phenotypes in mice [188–190]. Interestingly, genetic impairment of NMDAR function specifically within cortico-limbic inhibitory interneurons is sufficient to cause schizophrenia phenotypes [191], which is consistent with the observation that NMDAR blockers can result in suppressed activation of inhibitory interneurons, resulting in dis-inhibition of excitatory neurons [174]. This supports a role for decreased inhibition in schizophrenia phenotypes caused by reduced NMDAR function [192]. In addition to schizophrenia, impaired or diminished NMDAR function that results from genetic variants can be a cause of developmental encephalopathy involving epilepsy and intellectual disability [193].

Given the implication of NMDAR hypofunction in multiple brain diseases, NMDAR PAMs have been pursued as therapeutics, with numerous scaffolds serving as tool compounds [1, 131] (see Box 1). Multiple scaffolds exist for PAMs that alter NMDAR function independent of subunit composition. These relatively non-selective PAMs include the neurosteroids [194, 195], tetrahydroisoquinoline series [196], thienopyrimidin-4-ones [197], tetrahydrobenzothiophene series [198], as well as two individual compounds: GNE-9278 [199, 200] and PTC-174 [97, 201]. Moreover, a few of these series of PAMs have entered clinical trials, providing the first human data on actions of NMDA receptor potentiation. The NMDAR PAM CAD-9303 (similar to thienopyrimidin-4-ones) has been studied in schizophrenia patients (NCT04306146). The NMDAR PAM SAGE-718 [195], which is related to 24(S)-HC [202], is being studied in Huntington’s disease (Clinicaltrials.gov registry number: NCT05358821, NCT05107128), Parkinson’s disease (NCT05318937) and Alzheimer’s disease (NCT04602624). Additional PAMs (e.g. SGE-550) based on the 24(S)-HC scaffold have shown activity against genetic missense variants in genes encoding NMDAR subunits that diminish the function of the receptor [203]. While these first-generation NMDAR PAMs do not show selective affinity for specific NMDAR subunits, some do show differential effects on NMDARs with varied subunit composition. For example, the tetrahydroisoquinoline series, thienopyrimidin-4-ones, and PCT-174 enhance the maximal response of GluN2C- and GluN2D-containing NMDARs to a greater extent than GluN2B-containing receptors [97, 196, 197]. GluN2A-containing receptors show minimal enhancement of maximal responses with these PAMs, but do show changes in deactivation time course [197, 204].

### GluN2A PAMs

Genome wide association studies in schizophrenia have identified genes encoding synaptic proteins, including *GRIN2A* [184], which encodes GluN2A. Recent rare variant analysis also implicates *GRIN2A* as one of the top genes in which loss-of-function variants are associated with schizophrenia [183]. Therefore, enhancing GluN2A function could be an attractive therapeutic approach in schizophrenia. Interestingly, genetic variants in *GRIN2A* have also been identified in epilepsy aphasia syndromes [205–207]. These disease-associated variants include not only gain-of-function mutations that could directly over-activate excitatory neurons but also include loss-of-function variants, indicating that reduced NMDAR function could also lead to the network overactivation underlying epilepsy. The mechanism of loss-of-function variants in causing epilepsy and other phenotypes could involve reduced activation of NMDARs in inhibitory interneurons, leading to circuit disinhibition. A recent report suggests *GRIN2A* +/− mice, a model for patients with null variants, exhibit delayed hippocampal interneuron maturation, which could promote hypersynchronous activity [208].

Motivated by the clear implication of reduced *GRIN2A* function in disease, GluN2A-selective PAMs have been identified using high throughput screening, with examples including GNE-6901 and GNE-8324 [133]. These PAMs enhance the function of GluN1/GluN2A diheteromers or GluN1/GluN2A/GluN2B triheteromers, which are the predominant species of NMDARs located at the synaptic cleft in mature neurons [133]. GNE-6901 and GNE-8324 showed interesting differences in biophysical properties such as the degree to which glutamate potency was enhanced and receptor deactivation was slowed [133]. These PAMs also showed functional differences in brain slice neurophysiology experiments, with GNE-6901 enhancing NMDAR synaptic responses on both excitatory neurons and inhibitory interneurons, whereas GNE-8324 selectively enhanced NMDAR response on inhibitory interneurons but not excitatory neurons. The reason for this synaptic selectivity might involve differences in the microenvironment between synapses onto excitatory and inhibitory neurons that result in different susceptibility to potentiation by specific modes of PAM action, such as high ambient glutamate [209]. Alternatively, preferential enhancement of certain triheteromeric NMDARs such as those containing GluN2D in interneurons may contribute to this phenomenon.

Medicinal chemistry efforts on the earlier in vitro tool GluN2A PAMs resulted in useful in vivo tool compounds with improved bioavailability, pharmacokinetics, and brain penetration, as exemplified by GNE-0723 [210, 211]. Unlike GNE-8324, GNE-0723 was able to potentiate postsynaptic NMDAR currents in both inhibitory interneurons and excitatory neurons in recordings from brain slices [212]. Nonetheless, in vivo treatment with GNE-0723 normalized network hyperactivity and rescued cognitive function in mouse models of Alzheimer’s disease and Dravet syndrome that both have deficits driven by interneuron hypoactivity [212]. It may be that despite higher GluN2A expression in excitatory neurons compared to interneurons, NMDAR PAMs in general have stronger functional impacts on inhibitory vs. excitatory neuron activity in vivo because inhibitory interneurons exhibit high frequency firing and depolarized membrane potentials that could reduce Mg^2+^ block of NMDARs, allowing for a basal contribution of NMDAR synaptic currents to the drive of interneurons [192]. By comparison, excitatory neuron activity is relatively sparse [213], with NMDAR activation mostly occurring during rare coincident pre- and post-synaptic activity. Therefore, instead of having major effects on excitatory neuron activity, at least at low doses, NMDAR PAMs including GNE-0723 could predominantly enhance interneuron activity and shift the excitation-inhibition balance towards greater inhibition. Another potential mechanism of functional selectivity for interneurons could be GluN2A PAM effects on triheteromeric NMDARs containing both GluN2A and the GluN2D subunit, the latter which is expressed in interneurons and confers higher sensitivity to extrasynaptic glutamate [27]. As these PAMs increase glutamate potency, they could increase tonic NMDAR currents in interneurons and drive interneuron activation. At the same time at high PAM concentrations, amplification of NMDARs on excitatory neurons could become dominant and cause overactivation leading to seizures, as has been observed with AMPA PAMs [214]. Thus, achieving an adequate therapeutic index will be a critical challenge for NMDAR PAMs to be useful therapeutics.

### GluN2B PAMs

There are a number of lines of evidence to suggest that GluN2B PAMs may have effects on cognitive function. GluN2B overexpression in the forebrain in mice enhances synaptic plasticity and learning and memory, suggesting a potential role for GluN2B-containing NMDARs in memory formation [215–217], although these transgenic studies could involve compensatory mechanisms during development and other changes that might not be recapitulated by pharmacological approaches. Furthermore, GluN2B NAMs have schizophrenia-mimetic effects and disrupt cognition similar to NMDA channel blockers [159, 160], suggesting GluN2B potentiation may induce an opposing effect. While a number of pan-NMDAR PAMs can enhance GluN2B-containing receptors, relatively few small molecules are capable of selectively enhancing GluN2B activity. Two examples that are unlikely to lead to CNS therapeutics include polyamines such as spermine [218] and the aminoglycoside antibiotics neomycin, kanamycin, and tobramycin [219–221]. The aminoglycosides may increase glycine potency [219] without altering deactivation time course [220], consistent with a spermine-like mechanism of potentiation. Apart from these charged molecules, the identification of small, brain-penetrant drug-like molecules with GluN2B selectivity so far remains an elusive goal in medicinal chemistry programs.

### GluN2C and GluN2D PAMs

NMDARs that contain the GluN2C and GluN2D subunits have been historically understudied compared to NMDARs that contain GluN2A and GluN2B. Pharmacological tools to study these subunits began to emerge with the discovery of a series of competitive antagonists (e.g. UBP-141;[222, 223]) that initially showed modest selectivity for NMDARs containing GluN2D, but led to development of more selective compounds [224]. Work on GluN2D-containing receptors accelerated in 2010 and 2011 with the reports of GluN2C/D-selective PAMs (e.g. ( + )-CIQ) and NAMs (DQP-1105, QNZ-46; see [1]). These initial and subsequent GluN2C/D-selective tool compounds (e.g. NAB-14; [82] and (+)-EU1180-453; [225]) provide multiple means to explore GluN2C and GluN2D contribution to circuit and brain function. In addition, recognition that compounds such as PCT-174 [97] enhance responses of GluN2C- and GluN2D-containing NMDA receptors more than other subunits support the idea of functionally-derived selectivity, where potency is similar but maximal effect is subunit-dependent. The ability to differentially enhance GluN2C- and GluN2D-containing NMDARs over GluN2A- and GluN2B-containing receptors may reflect the relatively low basal open probability, allowing PAMs to produce greater fold potentiation of GluN2C- and GluN2D-containing NMDARs. So far only a single series of PAMs have been described that distinguish between GluN2C and GluN2D. The PYD series of PAMs bind at the interface of the NTD and ABD [226] and enhance receptor function through conformational selection, which involves modulator binding that stabilizes an active conformation of GluN1/GluN2C diheteromeric receptors [140, 143].

While the selectivity of these series is well-described for diheteromeric NMDARs that contain two copies of the GluN2 subunit, most (if not all) GluN2C and GluN2D are expressed as triheteromeric NMDARs [44]. On one hand this observation simplifies to some extent interpretation of pharmacological effects if no GluN2C or GluN2D diheteromeric receptors reach the plasma membrane. On the other hand, subunit-selective modulators likely have differential effects across the different triheteromeric assemblies. Pharmacological data exist for GluN2C/D PAMs (and NAMs) on triheteromeric assemblies GluN1/GluN2A/GluN2B, GluN1/GluN2A/GluN2C, GluN1/GluN2A/GluN2C, and GluN1/GluN2B/GluN2D [44, 97, 136], and various PAMs show different actions across these triheteromeric receptors. For example, the non-selective PAM PTC-174 potentiates GluN1/GluN2C and GluN1/GluN2D activity more than 10-fold and GluN1/GluN2B about 2-fold, but inhibits GluN1/GluN2A by about 0.5-fold [97]. The tetrahydroisoquinoline *S*-(-)-EU1180-55 similarly shows enhanced potentiation of GluN2C and GluN2D NMDARs over GluN2B. When evaluated in triheteromeric receptors, PTC- 174 potentiates GluN1/GluN2B/GluN2D and GluN1/GluN2A/GluN2C NMDARs to an intermediate level between GluN1/GluN2B and GluN1/GluN2D and between GluN1/GluN2A and GluN1/GluN2C receptors [97]. Interestingly, PTC-174 potentiates NMDAR-mediated responses in subthalamic nucleus neurons and hippocampal CA1 interneurons, but not hippocampal CA1 pyramidal neurons [97], suggesting the greater effect on GluN2B/GluN2D than GluN2B or GluN2A can bring about some functional selectivity.

Some GluN2A PAMs may have benefits in models featuring interneuron dysfunction, and we speculate above that this may be due to preferential effects of NMDAR PAMs in general on interneuron activity in vivo or preferential actions on the triheteromeric receptors expressed in interneurons. However, in terms of cellular expression in the cortex/hippocampus, GluN2A is actually higher on excitatory neurons compared to inhibitory interneurons (Fig. 3). By contrast, GluN2D is highly expressed in inhibitory interneurons [60, 89, 90], including PV interneurons. This could allow GluN2C/D-selective PAMs to have a preferential effect on inhibitory vs excitatory neurons, and perhaps therapeutic utility through alterations in GABAergic tone. GluN2C/D PAMs may be useful for improving anxiety behaviors as a recent study found enhanced anxiety- and depressive-like behaviors in mice with GluN2D reduced in the bed nucleus of the stria terminalis [84]. Additionally, subthalamic GluN2D expression is reduced in models of parkinsonism [227]. Further evaluation of these modulators is needed to fully delineate the utility in terms of subtype-dependence, activity-dependence, neuronal physiology, and neuronal circuit impact. These GluN2C- and GluN2D-selective PAMs might also impart therapeutic actions through regulation of GluN2C-containing NMDARs on astrocytes or on neurons in thalamus and other regions.

## Summary and future research directions

The therapeutic potential of NMDAR modulators in psychiatry has been a topic of interest for many years, given that NMDARs are involved in synaptic efficacy and plasticity. The recent decade has seen a renaissance in NMDAR pharmacology, driven in part by new knowledge gained from older agents, namely the channel blocker ketamine and the GluN2B NAMs. New classes of PAMs and NAMs with subunit-selectivity for nearly all the different NMDARs are being used to investigate the role of different receptor subtypes in specific neurological disease. This, in turn, is driving vigorous efforts to develop next generation subunit-selective agents to allow ever more precise targeting of circuit modification and that are suitable for clinical development. A major focus in the field continues to be linking macroscopic effects of these agents on behavior to underlying molecular mechanisms of action. Whereas the mechanism by which NMDAR inhibition produces a durable effect in depression is still not well-understood, uncovering the molecular and cellular basis for this effect, including the role of NMDAR subtypes, downstream signaling pathways, and the effects on other neurotransmitter systems could allow development of new antidepressants that act in new ways. Similar promise rests with subunit-selective positive allosteric modulators, which are being considered for a wide range of neuropsychiatric conditions. The expression of GluN2D subunits in interneurons offers a unique opportunity to sculpt interneuron activity and control overall network function. In addition, some of these new generation NMDAR modulators may be more effective when used in combination with other drugs, such as antipsychotics or antidepressants. Thus, future work needs to examine the therapeutic benefits of these combinations, as well as the potential for drug interactions and side effects. Finally, new technologies ranging from proteomics, high throughput sequencing, and neuroimaging techniques are poised to allow even deeper investigation of mechanisms of drug action, and thus should enhance selection of classes and new generations of NMDAR modulators for further development efforts.
